# Enhanced Biofilm Eradication and Reduced Cytotoxicity of a Novel Polygalacturonic and Caprylic Acid Wound Ointment Compared with Common Antiseptic Ointments

**DOI:** 10.1155/2021/2710484

**Published:** 2021-02-25

**Authors:** Bahgat Z. Gerges, Joel Rosenblatt, Y-Lan Truong, Ruth A. Reitzel, Ray Hachem, Issam I. Raad

**Affiliations:** Department of Infectious Diseases, Infection Control and Employee Health, The University of Texas MD Anderson Cancer Center, Houston, Texas, USA

## Abstract

Antiseptic wound ointments are widely used to treat dermal wounds that are microbially contaminated. Polygalacturonic acid (PG)+caprylic acid (CAP) is a novel combination that has been shown to eradicate biofilms. We developed a novel PG+CAP ointment and compared the biofilm eradication capability and cytotoxicity of PG+CAP with that of commercially available antiseptic wound ointments. We used a well-established biofilm model to quantitatively assess the eradication of organisms following exposure to the wound ointments for 2 hours. PG+CAP ointment completely eradicated *Candida albicans*, multidrug-resistant *Pseudomonas aeruginosa*, and methicillin-resistant *Staphylococcus aureus* biofilms, whereas MediHoney, polyhexamethylene biguanide (PHMB), and benzalkonium chloride (BZK) ointments failed to eradicate all biofilms within 2 hours. We assessed cytotoxicity by exposing L-929 fibroblasts to extracts of each ointment; Trypan blue exclusion was used to assess cell viability, and Alamar blue conversion was used to assess metabolic function. After exposure to PG+CAP and MediHoney, fibroblast viability was 96.23% and 95.23%, respectively (Trypan blue), and was comparable to untreated cells (98.77%). PHMB and BZK showed reduced viability (83.25% and 77.83%, respectively, *p* < 0.05). Metabolic activity results followed a similar pattern. Cytotoxicity of PG+CAP ointment towards erythrocytes was comparable to saline. PG+CAP ointment seems to be safe and can rapidly eradicate microbial biofilm; thus, PG+CAP ointment merits further in vivo testing as a potential antimicrobial wound ointment.

## 1. Introduction

Antiseptic wound ointments are widely used to treat dermal wounds that are microbially contaminated. These ointments play an important role in inhibiting microbial biofilms because the ointments are broad-spectrum and do not encourage the development of antibiotic-resistant microorganisms. Quaternary-ammonium benzalkonium chloride (BZK) wound ointment was shown to generate an adequate environment for wound healing; it improved cell proliferation and cell activity and suppressed the multiplication of bacteria [1, 2]. Polyhexamethylene biguanide (PHMB) has been shown to increase healing rates and reduce the incidence of wound infection [2]. Benzalkonium chloride and polyhexamethylene biguanide can be aggressive chemical agents whose use on delicate tissues in wound beds can be accompanied by adverse effects [3]. Antibiotics are an alternative to antiseptic ointments because of their lower toxicities, but the wide use of antibiotics has led to the development of antibiotic-resistant organisms, including some that are resistant to multiple drugs [4]. In addition, biofilms are particularly resistant to antibiotics because of their extracellular matrix polysaccharides, which can restrict the diffusion of antibiotics [5].

As alternatives to both antiseptic and antibiotic ointments, natural plant-based agents have been shown to provide optimal biofilm disinfection without leading to antimicrobial resistance [6]. MediHoney [7] has been successfully used to treat recalcitrant wounds [8–12], wounds in neonates [13], and wound infection in severely immunocompromised patients [7], but it has also been ineffective in other studies [14]. The combination of polygalacturonic acid (PG) and caprylic acid (CAP) has been shown to synergistically eradicate biofilms in an in vitro model of typical hospital and foodborne infectious pathogenic biofilms (methicillin-resistant *Staphylococcus aureus* (MRSA), multidrug-resistant *Pseudomonas aeruginosa*, *Candida albicans*, *Escherichia coli*, and *Salmonella enteritidis*) [6]. This study provided the foundation for preparing an optimized PG+CAP ointment formulation that was studied here. In the current study, we compared the biofilm eradication capability and cytotoxicity of the novel PG+CAP ointment with that of antiseptic ointments containing benzalkonium chloride, polyhexamethylene biguanide, and MediHoney.

## 2. Material and Methods

### 2.1. Materials

Benzalkonium chloride (BZK, BlastX, Next Science, Jacksonville, FL), polyhexamethylene biguanide (PHMB, Prontosan, B. Braun, Bethlehem, PA), and MediHoney (Integra Life Sciences, Princeton, NJ) wound ointments were purchased and used directly, while PG+CAP ointment was prepared in the laboratory as previously described [6]. The ointment base for PG+CAP was an aqueous gel containing 2-hydroxyethylcellulose and glycerol.

### 2.2. Biofilm Eradication Assay

Biofilm eradication testing was conducted using highly virulent clinical isolates of MRSA (MDA #120), multidrug-resistant *P. aeruginosa* (MDA #118), and *C. albicans* (MDA #117), as representative hospital-acquired infection pathogens from cancer patients. For testing, the organisms were grown from glycerol stock on trypticase soy agar+5% sheep blood (for bacteria) or on Sabouraud dextrose agar (*C. albicans*). Each organism was inoculated into Muller Hinton broth and diluted to 0.5 McFarland. Further dilutions were made as necessary for testing.

A well-established biofilm colonization model was employed to test eradication of pathogenic biofilms following 2 hours of exposure to different ointments [6]. Briefly, 1 cm silicone disks were placed in 24-well flat-bottom cell culture plates and exposed to 1 mL of human plasma overnight at 37°C. Biofilm was established on silicone disks by inoculating with the challenge organism (1 mL of 5.5 × 10^5^ CFU/mL) and incubating at 37°C for 24 hours. All culture liquid was then removed, and disks were washed for 30 minutes in isotonic sterile saline to remove any remaining planktonic organisms. After washing, disks were exposed to ointments, control ointment base, and control by adding Muller Hinton broth and incubating at 37°C for 120 minutes. After exposure, viable organisms remaining on the surface of the silicone disks were assessed by disrupting biofilm via sonicating the disks in 5 mL of isotonic saline for 15 minutes. The resulting sonicate was serially diluted and quantitatively cultured onto trypticase soy agar+5% sheep blood for *Staphylococcus aureus* and *P. aeruginosa* or on Sabouraud dextrose agar for *C. albicans*. Each ointment for each organism was tested with six replicates. To ensure eradication was complete (no surviving dormant or persister cells) from biofilms for which no viable colonies were recovered following the exposure to ointments, we conducted regrowth experiments by first exposing biofilm-colonized disks to each experimental solution, then rinsing, and subsequently transferring the disks to fresh broth and reincubating for an additional 24 hours. Following the 24-hour regrowth interval, disks were sonicated and cultured as indicated above to determine whether any organisms remaining embedded in the biofilm were still viable.

### 2.3. Cytotoxicity Tests

NCTC clone 929 areolar fibroblast mouse (*Mus musculus*) cells were used in our study. Cell culture was performed according to fibroblast protocols as described by Rosenblatt et al. [6] and de Gomes et al. [15]. PG+CAP in ointment base, BZK, PHMB, MediHoney, and nonantimicrobial ointment base were tested for cytotoxicity by Alamar blue and Trypan blue exclusion assays. Briefly, cells were incubated at 37°C with 5% CO_2_ until the cells formed a monolayer (60% confluent); then, a 2% extract of each ointment was added to its corresponding wells and incubated at 37°C with 5% CO_2_ for 24 hours. In the Trypan blue exclusion test, live cells with intact membranes exclude the Trypan blue dye whereas dead cells do not, and therefore, dead cells are stained with blue cytoplasm [16]. Live and dead cells were counted using a hemocytometer. The Alamar blue cell viability assay (Life Technologies Corp., Carlsbad CA, United States) assessed the metabolic activity of fibroblasts following 24 hr exposure to the antiseptic ointments and controls [17]. After 24 hours of ointment exposure, medium was replaced with 200 *μ*L HBSS+10% Alamar blue reagent and incubated at 37°C with 5% CO_2_ for 4 hours. Cell viability (absorbance) was determined at 570 nm using a microplate reader spectrophotometer, and the absorbance for each ointment was compared with that of controls.

Acute cytotoxicity towards bovine erythrocytes was assessed by preparing 2% extracts in whole bovine blood (Lampire Biological Laboratories, Pipersville, PA), incubating for 24 hrs, and then counting viable erythrocytes from 1 : 200 dilution in normal saline using a hemocytometer. Results from 5 replicates were collected for each ointment. 2% saline in whole blood was used as a control.

### 2.4. Statistical Analyses

To determine whether there was a significant difference between ointments and controls in the assays, the Kruskal-Wallis test was used. Pairwise comparisons were assessed using the Mann-Whitney *U* test to compare the performance of comparators. All tests were two-sided with an alpha level of 0.05. *p* < 0.05 was used to determine significance.

## 3. Results

### 3.1. Biofilm Eradication

**Figure 1:**
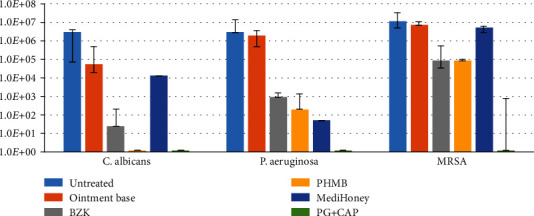
Eradication of biofilms from representative infectious pathogens *Candida albicans*, multidrug-resistant *Pseudomonas aeruginosa*, and methicillin-resistant *Staphylococcus aureus* (MRSA) by 1% polygalacturonic acid+0.4% caprylic acid (PG+CAP) wound ointment compared with commercially used ointments after 2 hours of exposure. Nonantimicrobial ointment base and nontreated disks were used as controls. Data are presented as the median recovered viable colonies; bars indicate the range.

Figure 1 presents the results of the biofilm eradication experiment. PG+CAP was able to completely eradicate all tested pathogens after 2 hours of incubation. PHMB was able to eradicate *C. albicans* but was not able to fully eradicate biofilms of *Staphylococcus aureus* and *P. aeruginosa*. BZK and MediHoney did not fully eradicate biofilms of any of the tested pathogens. For MRSA, PG+CAP was significantly more efficacious than PHMB (*p* = 0.02), BZK (*p* = 0.002), and MediHoney (*p* = 0.002) in eradicating biofilms. PG+CAP eradicated significantly more *P. aeruginosa* biofilms than did BZK (*p* = 0.015) and significantly more *C. albicans* biofilm compared with MediHoney (*p* = 0.015). No growth was observed in the regrowth experiments for any ointments for which no viable colonies were recovered in the biofilm eradication assay, verifying that eradication was complete.

### 3.2. Cytotoxicity

**Figure 2:**
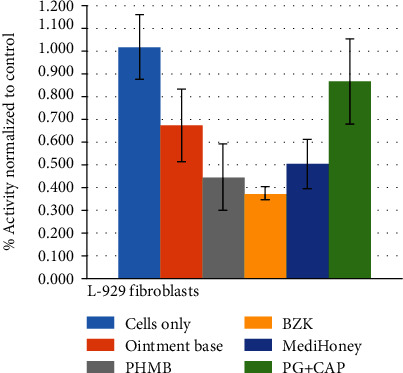
In vitro cytotoxicity testing by Alamar blue metabolic activity assay. L-929 fibroblasts were treated with a 2% extract of 1% polygalacturonic acid+0.4% caprylic acid (PG-CAP), MediHoney, BZK, or PHMB. Nonantimicrobial ointment base and untreated cells were used as controls. Results are expressed as a percentage of absorbance relative to control, as measured by a spectrophotometer at 570 nm. Higher activity indicates lower cytotoxicity.

Table 1 shows the results of Trypan blue exclusion tests. Statistically, mean viable cells of PG+CAP and MediHoney were comparable to untreated cells while PHMB and BZK had reduced viability (*p* < 0.05). Results of the Alamar blue metabolic activity assay are shown in Figure 2. Fibroblasts treated with MediHoney, PHMB, and PG+CAP had comparable metabolic activity compared to untreated cells while metabolic activity with BZK treatment was reduced (*p* < 0.05).  Table 2 shows the average number of viable erythrocytes and standard deviations from the blood cytotoxicity testing.

## 4. Discussion

Of the ointments assessed, PG+CAP was uniquely able to rapidly eradicate all representative biofilms without producing significant cytotoxicity relative to nonantimicrobial controls. The conventional antiseptic ointments (BZK and PHMB) were more cytotoxic and less broad-spectrum than PG+CAP. Previous studies reported that BZK was cytotoxic to fibroblasts, keratinocytes, and other epithelial cells [18]. Electron microscopy analysis revealed damage to subcellular organelles [19]. Further studies suggest that BZK induced oxidative stress in mitochondria leading to production of reactive oxygen molecules than can lead to irritation [20]. Our results showed that BZK failed to rapidly eradicate biofilms of the tested pathogenic isolates. Increased use of quaternary-ammonium- (QAC-) based biocides and disinfectants such as BZK has led to concern about reduced bacterial susceptibility to treatment and biocide effectiveness [21, 22]. Long-term exposure of microbial communities to QACs increased selection for both QAC-resistant and antibiotic-resistant bacteria [23, 24].

Fibroblasts cells treated with PHMB generated discernable cytotoxicity in our study. These results are consistent with previous studies with PHMB on epithelial cells [25]. Mechanistic studies on PHMB concluded that it induced inflammatory responses through activation of nuclear factor kappa B activation and its signaling pathway [26]. The greater cytotoxicity of BZK ointment relative to PHMB ointment in our experiment was comparable to a previous study on the bioactive compounds [27]. MediHoney was less cytotoxic than BZK and PHMB and comparable to PG+CAP; however, MediHoney produced a greater reduction of normal metabolic activity relative to PG+CAP. Furthermore, MediHoney was not able to rapidly eradicate biofilms of any of the tested pathogens potentially indicating less antimicrobial activity against wound biofilms. The cytotoxicity of PG+CAP and MediHoney towards erythrocytes was essentially the same as for saline. BZK and PHMB produced greater toxicity towards erythrocytes than saline but were comparable to each other.

Dermal wounds have been reported to exhibit improved healing at acidic pH [28]. The PG+CAP ointment used in our study had a pH of 4.25. In the ionized (deprotonated) state (neutral pH), the caprylate ion is a nutrient with a well-established metabolic profile in mammals [29]. CAP can become ionized to a benign nutrient when the pH rises above 4.8 [30]. PG has been widely used in hydrocolloid wound dressings, with the reported benefits of maintaining a moist, acidic environment and providing a bacterial barrier [31]. PG naturally maintains a pH in the same range as honey. This together with the antimicrobial effects with CAP described above supports the synergistic use of PG+CAP as a promising ointment for the treatment of wounds.

These findings suggest that PG+CAP ointment is both safe and can rapidly eradicate microbial biofilm; thus, PG+CAP ointment merits further in vivo testing as a potential antimicrobial wound ointment with low toxicity for treating biofilm contaminated wounds. In this regard, future ultrastructural analysis of biofilms by electron microscopy following treatment with PG+CAP ointment would also be worthwhile.

## Figures and Tables

**Table 1:** In vitro cytotoxicity assessment of cell viability for 1% polygalacturonic acid+0.4% caprylic acid (PG+CAP) wound ointment compared with commercially used ointments after exposure of L-929 fibroblasts to a 2% extract for 24 hours, using the Trypan blue exclusion method.

Treatment	Mean cells/mL ± standard deviation		% viable^∗^
	Live cells	Dead cells	
Untreated cells	1.77 ± 0.58 × 10^6^	2.3 ± 0.58 × 10^4^	98.77%
Base ointment	1.59 ± 0.67 × 10^6^	4.4 ± 0.63 × 10^4^	97.15%
PG+CAP	1.35 ± 2.06 × 10^6^	5.4 ± 3.7 × 10^4^	96.29%
BZK	1.29 ± 3.76 × 10^6^	2.05 ± 12.16 × 10^5^	77.83%
PHMB	1.32 ± 5.09 × 10^6^	2.38 ± 16.74 × 10^5^	83.25%
MediHoney	1.49 ± 2.31 × 10^6^	6.7 ± 4.76 × 10^4^	95.23%

^∗^Cell viability is expressed as the percentage of viable cells relative to total cells.

**Table 2:** In vitro cytotoxicity assessment of erythrocyte viability following 24-hour exposures to PG+CAP and commercially used ointments based on dilution and counting with a hemocytometer. Results for each ointment presented are averages ± standard deviation for 5 replicates.

Treatment	Viable erythrocytes (average ± standard deviation)
Saline control	3.82 ± 0.22 × 10^6^
Base ointment	3.81 ± 0.16 × 10^6^
PG+CAP	3.82 ± 0.20 × 10^6^
BZK	3.48 ± 0.10 × 10^6^
PHMB	3.49 ± 0.55 × 10^6^
MediHoney	3.81 ± 0.32 × 10^6^

## Data Availability

Data is available on request.
